# Molecular Evolution of the Transmembrane Domains of G Protein-Coupled Receptors

**DOI:** 10.1371/journal.pone.0027813

**Published:** 2011-11-21

**Authors:** Sarosh N. Fatakia, Stefano Costanzi, Carson C. Chow

**Affiliations:** Laboratory of Biological Modeling, National Institute of Diabetes and Digestive and Kidney Diseases, National Institutes of Health, Bethesda, Maryland, United States of America; University of Wyoming, United States of America

## Abstract

G protein-coupled receptors (GPCRs) are a superfamily of integral membrane proteins vital for signaling and are important targets for pharmaceutical intervention in humans. Previously, we identified a group of ten amino acid positions (called key positions), within the seven transmembrane domain (7TM) interhelical region, which had high mutual information with each other and many other positions in the 7TM. Here, we estimated the evolutionary selection pressure at those key positions. We found that the key positions of receptors for small molecule natural ligands were under strong negative selection. Receptors naturally activated by lipids had weaker negative selection in general when compared to small molecule-activated receptors. Selection pressure varied widely in peptide-activated receptors. We used this observation to predict that a subgroup of orphan GPCRs not under strong selection may not possess a natural small-molecule ligand. In the subgroup of MRGX1-type GPCRs, we identified a key position, along with two non-key positions, under statistically significant positive selection.

## Introduction

G protein-coupled receptors (GPCRs) constitute a diverse superfamily of integral membrane proteins involved in intercellular signal transduction. Their genes are expressed in almost all eukaryotes [1], [2], [3], [4], [5]. The receptor consists of a single polypeptide chain that loops through the cell membrane seven times to form an interhelical cavity of seven alpha-helical transmembrane domains (7TMs). GPCRs are the largest superfamily of integral membrane proteins in humans. About half of the GPCRs in the human genome are non-olfactory receptors [6], [7], [8]. These receptors mediate vital physiological functions and are a major target for pharmaceutical interventions [9], [10]. Although diverse in sequence composition and function, GPCRs share a common molecular architecture of 7TMs connected via three intracellular and three extracellular loops. Fredriksson and Schioth have categorized the GPCRs into five distinct families [8], [11] - Glutamate (also known as class C), Rhodopsin (also known as class A), Adhesion, Secretin (collectively known as class B) and Frizzled/Taste (also known as class F). Nearly 85% of the non-olfactory receptors belong to class A. Class A receptors bind different natural ligands that range from small-molecules such as ADP to larger ones such as neuropeptides or chemokines.

New protein functions in paralogous protein superfamilies arise by the modulation of older existing ones [12]. During this evolutionary process, some of the amino acid residues remain conserved. However, mutations of some residues may be followed by compensatory mutations elsewhere to preserve function or give rise to new ones. The identification of such related residue positions can help to identify biologically relevant sets of residues in protein superfamilies. Previously, we identified a set of positions in the interhelical cavity enclosed within the 7TM domain of class A GPCRs that have high mutual information (MI) with other positions and each other [13], [14]. These key positions were found to be located in the region that constitutes the binding cavity of GPCRs whose structures have been solved. Biochemical data suggest that this region hosts the orthosteric binding cavity for all class A GPCRs naturally activated by small molecules.

Here, we examine the nucleotide sequences corresponding to these GPCRs to probe the evolutionary selection pressure at these key positions. Synonymous nucleotide substitutions (‘silent’ mutations) do not change the translated amino acid sequence so their substitution rate *d*_S_ (also referred to as K_S_) is not subject to selective pressure on the expressed protein. Nonsynonymous mutations alter the amino acid sequence and their substitution rate *d*_N_ (also referred to as K_A_) is a function of selective pressure on the protein. The ratio *d*_N_/*d*_S,_, referred to as ω, gives a measure of the selection pressure at that site [15], [16]. When there exists negative or purifying selection pressure at a codon position, ω<1 and synonymous substitutions dominate. When the position is under positive or adaptive selection, ω>1 and nonsynonymous substitutions dominate. Rare instances of positive selection are of special interest in tracing functional divergence among protein families and physiological adaptations in humans [17], [18], [19], [20], [21]. When the position evolves neutrally – without any strong preferential selection, the two substitution rates are nearly equal. Here we determine ω at the key positions and compare it to other 7TM positions. If the selection pressure at the key positions is less neutral then on other positions then this supports the hypothesis that the high mutual information between the key positions and associated high entropy did not simply arise from evolutionary drift.

## Results

All subgroups of human GPCRs were classified into three categories in terms of their natural ligands: 1) small molecules (including biogenic amines, nucleosides and nucleotides), 2) lipids, and 3) peptides. GPCR subgroups whose natural ligands could not be exclusively classified as any of the above were categorized as divergent. A number of human GPCRs are orphans with no known natural ligands. The list of GPCR subgroups and the chemical class of associated natural ligands is in Tables 1, 2, 3 and 4. Of the 45 subgroups of GPCRs, excluding subgroup 13b, 10 subgroups are activated by small molecules listed in Table 1, 9 subgroups are activated by lipids listed in Table 2, and 19 subgroups are activated by peptides listed in Table 3. Six subgroups were categorized as divergent, because they are activated by natural ligands that belong to different chemical classes or contain two or more orphans. One subgroup exclusively contained human orphan GPCRs. The divergent and orphan subgroups are listed in Table 4.

**Table 1:** List of class A GPCRs included in the study.

Subgrp idx	# GPCRs in subgrp	GPCRs included in the subgroups^a^	Natural ligand	Chemical class of natural ligand^b^	Notes^c^
1	5	CHRM1 (ACM1), CHRM2 (ACM2), CHRM3 (ACM3), CHRM4 (ACM4), CHRM5 (ACM5)	acetylcholine	small	
2	5	DRD1, DRD2, DRD3, DRD4, DRD5	dopamine	small	
3	5	P2RY12 (P2Y12), P2RY13 (P2Y13), P2RY14 (P2Y14), GPR87, **GPR171** (**GP171**)	nucleotides, lysophosphatidic acid (GPR87)	small	o, S
4	7	HTR1A (5HT1A), HTR1B (5HT1B), HTR1D (5HT1D), 5HT1F (HTR1F), HTR1E (5HT1E), HTR5A (5HT5A), HTR7 (5HT7R)	5-hydroxytryptamine	small	
5	5	P2RY1, P2RY2, P2RY4, P2RY6, P2RY11 (P2Y11)	nucleotides	small	
6	3	MTNR1A (MTR1A), MTNR1B (MTR1B), **GPR50** (**MTR1L**)	melatonin	small	o
7	5	ADRA1A (ADA1A), ADRA1B (ADA1B), ADRB1, ADRB2, ADRB3	Adrenaline	small	
8	3	HTR2A (5HT2A), HTR2B (5HT2B), HTR2C (5HT2C)	5-hydroxytryptamine	small	
9	4	HRH1, HRH2, HRH3, HRH4	Histamine	small	
10	3	ADORA1 (AA1R), ADORA2A (AA2AR), ADORA2B (AA2BR)	Adenosine	small	

a The receptors are indicated through their gene name. Uniprot name, when different from the gene name, and common synonyms are listed in parentheses. Orphan receptors are indicated in bold and indicated as ‘o’ in Notes.

b Small indicates “small molecules” and refers to biogenic amines, nucleosides and nucleotides.

c The symbol “o” indicates that the subgroup has one or more orphan GPCR.

**Table 2:** List of class A GPCRs included in the study (continued from Table 1).

Subgrp idx	# GPCRs in subgrp	GPCRs included in the subgroups^a^	Natural ligand	Chemical class of natural ligand	Notes^c^
11	6	S1PR2 (EDG5), S1PR1 (EDG1), S1PR3 (EDG3), S1PR5 (EDG8), LPAR1 (EDG2), LPAR3 (EDG7)	sphingosine 1-phosphate, lysophosphatidic acid (LPAR1, LPAR3)	lipid	
12	3	GPR3, GPR6, GPR12	sphingosine 1-phosphate	lipid	
13	3	FFAR1 (GPR40), FFAR2 (GPR43), FFAR3 (GPR41)	free fatty acids	lipid	
14	7	PTGDR (PD2R), PTGER1 (PE2R1), PTGER3 (PE2R3), PTGER4 (PE2R4), PTGFR (PF2R), PTGIR (PI2R), TBXA2R (TA2R)	prostaglandins, thromboxane (TA2R)	lipid	
15	3	CYSLTR1 (CLTR1), CYSLTR2(CLTR2), GPR17	cysteinyl leukotrienes	lipid	
13b^d^	4	FFAR1 (GPR40), FFAR2 (GPR43), FFAR3 (GPR41), GPR42 (pseudogene)	free fatty acids	lipid	
16	5	LPAR4 (P2RY9), LPAR6 (P2RY5), **GPR174** (**GP174**), P2RY10 (P2Y10), PTAFR	lysophosphatidic acid, sphingosine 1-phosphate (P2Y10), platelet activating factor	lipid	o
17	5	RRH (OPSX), OPN3, OPN4, OPN5, RGR	Retinoids	lipid	
18	4	OPN1MW (OPSG), OPN1LW (OPSR), RHO (OPSD), OPN1SW (OPSB)	Retinoids	lipid	
19	3	GPR81, GPR109B (G109B), GPR109A (G109A)	hydroxylated short and medium-chain fatty acids	lipid	

a The receptors are indicated through their gene name. Uniprot name, when different from the gene name, and common synonyms are listed in parentheses. Orphan receptors are indicated in bold and indicated as ‘o’ in Notes.

c The symbol “o” indicates that the subgroup has one or more orphan GPCR.

d Derived from subgroup 13 through the addition of the pseudogene GPR42.

**Table 3:** List of class A GPCRs included in the study (continued from Tables 1, 2).

Subgrp idx	# GPCRs in subgrp	GPCRs included in the subgroups^a^	Natural ligand	Chemical class of natural ligand^b^	Notes^c^
20	3	TACR1 (NK1R), TACR1 (NK2R), TACR3 (NK3R)	tachykinin neuropeptides	peptide	
21	3	TSHR, LHCGR (LSHR), FSHR	glycoprotein hormones	peptide	
22	4	F2R (PAR1), F2RL1 (PAR2), F2RL2 (PAR3), F2RL3 (PAR4)	unmasked N-terminus	peptide	
23	5	**GPR83**, NPY1R, NPY2R, PPYR1 (NPY4R), NPY5R	neuropeptide Y and peptide YY	peptide	o
24	3	C3AR1 (C3AR), C5AR1 (C5AR), GPR77 (C5ARL)	anaphylatoxins	peptide	
25	4	EDNRA, EDNRB, **GPR37**, **GPR37L1** (**ETBR2**)	Endothelins	peptide	o
26	5	**LGR5**, **LGR6**, RXFP1 (LGR7), RXFP2 (LGR8)	Relaxin	peptide	
27	3	GALR1, GALR2, GALR3	Galanin	peptide	N
28	4	OPRL1 (OPRX), OPRM1 (OPRM), OPRD1 (OPRD), OPRK1 (OPRK)	opioid peptides	peptide	N
29	3	SSTR2 (SSR2), SSTR3 (SSR3), SSTR5 (SSR5)	somatostatins	peptide	
30	3	GRPR, NMBR, BRS3	bombesin-related peptides	peptide	
31	3	MC3R, MC4R, MC5R	melanocortins	peptide	N
32	3	AVPR1A (V1AR), AVPR1B (V1BR), AVPR2 (V2R)	Vasopressin	peptide	N
33	10	CXCR1, CXCR2, CXCR3, CXCR4, CXCR5, CXCR6, CCR6, CCR7, CCR9, CCR10	Chemokines	peptide	
34	5	APLNR (APJ), AGTR1 (AG2R, AG2S), RL3R1 (RLN3R2), RXFP4 (RLN3R2)	apelin (APLNR), angiotensin (AGTR1), relaxin (RL3R1, RLN3R2)	peptide	
35	3	NTSR1 (NTR1), NTSR2 (NTR2), GPR39	neurotensin, obestatin (GPR39)	peptide	
36	9	CCR1, CCR2, CCR3, CCR4, CCR5, CCR8, CCRL2, CX3CR1(CX3CR1, C3X1), CCBP2	Chemokines	peptide	S
37	3	FPR1, FPR2 (FPRL1), FPR3 (FPRL2)	N-formyl-methionyl peptides (FPRs)	peptide	
38	4	MRGPRX1 (MRGX1), MRGPRX2 (MRGX2), **MRGPRX3** (**MRGX3**), **MRGPRX4** (**MRGX4**)	enkephalins (MRGPRX1), cortistatins (MRGPRX2)	peptide	o

a The receptors are indicated through their gene name. Uniprot name, when different from the gene name, and common synonyms are listed in parentheses. Orphan receptors are indicated in bold and indicated as ‘o’ in Notes.

c The Symbol “N” indicates that pairs of receptors of the subgroup do not satisfy max(*d*_N_)<1 in the Nei-Gojobori counting scheme [64]. The symbol “S” indicates that pairs of receptors from the subgroup do not satisfy max (*d*_S_)<3 in the Nei-Gojobori scheme. The symbol “o” indicates that the subgroup has one or more orphan GPCR.

**Table 4:** List of class A GPCRs included in the study (continued from Tables 1, 2 and 3).

Subgrp idx	# GPCRs in subgrp	GPCRs included in the subgroups^a^	Natural ligand	Chemical class of natural ligand^b^	Notes^c^
39^e^	5	**GPR101** (**GP101**), **GPR161** (**GP161**), **GPR135** (**GP135**), GPR63, **GPR45**	sphingosine 1-phosphate	divergent	o
40	3	GPR4, GPR65 (PSYR), GPR68 (OGR1)	protons (GPR4 and GPR68), glycosphingolipids (GPR65)	divergent	N
41	4	MAS1 (MAS), **MAS1L** (**MRG**), MRGPRD (MRGRD), **MRGPRF** (**MRGRF**, **GPR140**)	angiotensin (MAS1), β-alanine (MRGRD)	divergent	o
42^e^	5	TAAR1 (TAR01), **TAAR5**, **TAAR6** (**TAR4**), **TAAR8** (**TAR5**), **TAAR9** (**TAR3**)	trace amines	divergent	o,S
43^g^	10	C3AR1 (C3AR), C5AR1 (C5AR), GPR77 (C5ARL), CMKLR1(CML1), FPR1, FPR2 (FPRL1), FPR3 (FPRL2), GPR1, GPR32, GPR44 (CRTH2)	Anaphylatoxins (C3AR1, C5AR1, GPR77), chemokines (CMKLR1), N-formyl-methionyl peptides (FPRs), chemerin (GPR1), resolvins (GPR32), prostanoids (GPR44)	divergent	
44^f^	8	MAS1 (MAS), **MAS1L** (**MRG**), MRGPRD (MRGRD), **MRGPRF** (**MRGRF**, **GPR140**), MRGPRX1 (MRGX1), MRGPRX2 (MRGX2), **MRGPRX3** (**MRGX3**), **MRGPRX4** (**MRGX4**)	angiotensin (MAS1), β-alanine (MRGRD), enkephalins (MRGPRX1), cortistatins (MRGPRX2)	divergent	o
45	3	**GPR27**, **GPR85**, **GPR173**		orphans	o

a The receptors are indicated through their gene name. Uniprot name, when different from the gene name, and common synonyms are listed in parentheses. Orphan receptors are indicated in bold and indicated as ‘o’ in Notes.

c The Symbol “N” indicates that pairs of receptors of the subgroup do not satisfy max(*d*_N_)<1 in the Nei-Gojobori counting scheme [64]. The symbol “S” indicates that pairs of receptors from the subgroup do not satisfy max (*d*_S_)<3 in the Nei-Gojobori scheme. The symbol “o” indicates that the subgroup has one or more orphan GPCR.

e Listed within the category of divergent receptors because only one member is not an orphan receptor.

f Group derived by the merging of groups 38 and 41.

g Contains also the three N-formyl-methionyl peptide receptors listed in subgroup 37.

**Figure 1:**
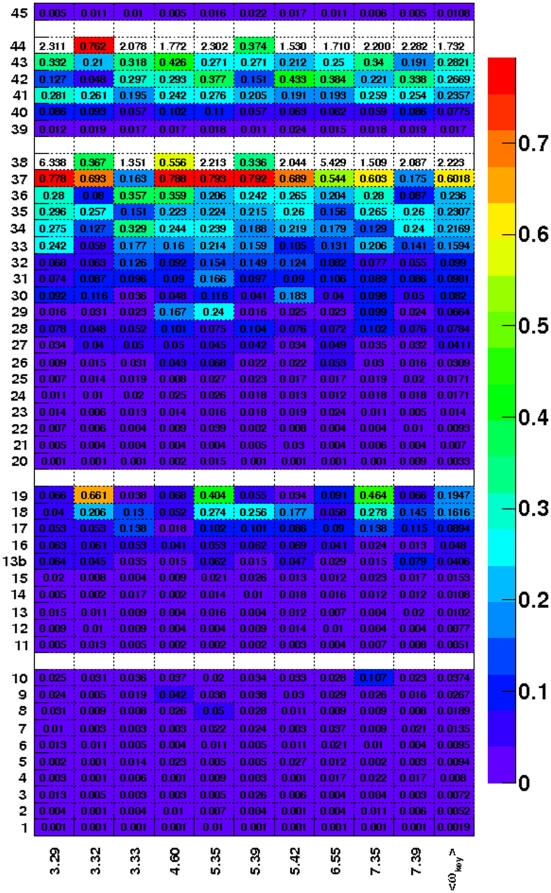
The ω_key_ values at key positions of subgroups of class A non-olfactory human GPCRs. Columns 1–10 represent the computed ω_key_ at the 10 key class A positions listed along the X axis using the Ballesteros-Weinstein index for GPCRs. The color code represents ω values ranging from violet (ω∼10^−3^) to red (ω∼1). GPCRs from forty-five different subgroups (labeled 1–45) are listed in Tables 1, 2, 3 and 4. Subgroups 1–10 are receptors that are naturally activated by small molecules (Table 1), 11–19 by lipids (Table 2), 20–38 by peptides (Table 3). Subgroups 39–44 are categorized as divergent and subgroup 45 exclusively contains orphan GPCRs (Table 4).

The ω values were determined for subgroups with at least three paralogs. Selection pressure at the key positions, ω_key_, is shown in Figure 1. The ω_key_ and its average, <ω_key_>, of subgroups associated with small molecules differed from that of subgroups associated with lipids and peptides. The Kruskal-Wallis rank sum test showed that <ω_key_> for small molecule-activated receptors had significantly lower values compared to subgroups of lipid-activated receptors, peptide-activated receptors and divergent receptors (p<0.003). The ω_key_ values from all ten subgroups activated by small molecules showed strong negative selection (ω<0.05).

We confirmed that human MRGX1-type receptors are under positive selection [22], [23]. Positive selection at three positions was inferred in subgroup 38 (MRGX1, MRGX2, MRGX3 and MRGX4 pain receptors) using three different tests. The results of the likelihood ratio estimates are shown in Table 5. The results of ω for key positions and positions with posterior probability of positive selection exceeding 0.5 are shown in Table 6. We inferred strong positive selection at key position 3.29 in the Ballesteros-Weinstein scheme [24], (ω = 6.3, posterior probability for ω>1 = 0.998). Two non-key positions: 2.56 (ω = 6.1, posterior probability for ω>1 = 0.948) and 2.60 (ω = 6.1, posterior probability for ω>1 = 0.947) were also under positive selection. Six of the key positions (5.35, 3.33, 5.42, 6.55, 7.35 and 7.39) were not under statistically significant positive selection. Three key positions (3.32, 4.60 and 5.39) were under negative selection. Subgroup 41 (MAS1L, MRGRD, MAS, and MRGRF pain receptors) did not show any statistically significant signature for positive selection. Previous studies had demonstrated positive selection pressure for the combined subgroups 41 and 38 (MRG receptors from humans and model organisms) [22], [23]. We inferred that the combined subgroup, 44, also exhibited positive selection exclusively within 7TMs but subgroup 41 did not exclusively exhibit statistically significant positive selection. Results from the likelihood ratio test for subgroup 44 are included in Table 5. An independent analysis of subgroup 44 confirmed statistically significant positive selection at key positions 3.29 and 5.35 along with two non-key positions 2.57 and 2.60. (Position 2.60 showed positive selection in subgroup 38 but not position 2.57).

**Table 5:** P-value and likelihood ratio (*LR*) estimates from three PAML strategies for subgroups 38 and 44.

PAML nestedmodel pairs	subgroup 38		subgroup 44	
	Δ = *ln*(L_Alt_/L_Null_) = *ln*L_Alt_−*ln*L_Null_	P-value	Δ = *ln*(L_Alt_/L_Null_) = *ln*L_Alt_−*ln*L_Null_	P-value
Test 1 (M2a vs. M1a)	8.90	<5.0×10^−4^	7.71	<2.5×10^−3^
Test 2 (M8 vs. M7)	8.94	<5.0×10^−4^	17.65	<<5.0×10^−4^
Test 3 (B vs. A)	7.98	<5.0×10^−3^	31.82	<<5.0×10^−4^

Result of **Δ** and P-value from Tests 1, 2 and 3. ***LR*** **= 2Δ** = 2*ln* (L_Alt_/L_Null_) = 2(*ln*L_Alt_−*ln*L_Null_).

**Table 6:** ω for subgroup 38.

7TM MSA position index	key	Ballesteros-Weinstein index	posterior probability (ω>1)	NEB ω	comment
8		1.37	0.562	3.953	-
19		1.48	0.680	4.598	-
***49***		***2.56***	***0.948***	***6.065***	***Positive***
50		2.57	0.610	4.226	-
***53***		***2.60***	***0.947***	***6.062***	***Positive***
57		2.64	0.595	4.147	-
61		3.22	0.514	3.690	-
62		3.23	0.824	5.390	-
64		3.25	0.794	5.228	-
65		3.26	0.533	3.796	-
***68***	***X***	***3.29***	0.998**	***6.338***	***Positive***
69		3.30	0.531	3.783	-
71	X	3.32	0.001	0.367	Negative
72	X	3.33	0.094	1.351	-
77		3.38	0.776	5.126	-
110		4.56	0.930	5.967	-
114	X	4.60	0.010	0.556	Negative
117	X	5.35	0.253	2.213	-
121	X	5.39	0.001	0.336	Negative
124	X	5.42	0.215	2.044	-
168	X	6.55	0.831	5.429	-
171	X	7.35	0.127	1.509	-
175	X	7.39	0.222	2.087	-
179		7.43	0.574	4.022	-

Model M8 NEB values obtained from subgroup 43. Key position 3.29 is under positive selection (** denotes statistically significant posterior probability for ω>1). Two non-key positions, 2.56 and 2.60, have posterior probability exceeding 90% for positive selection. All positions with posterior probability for ω>1which exceed 0.5 are represented. Results of ω from the 10 key positions are also included. Key positions identified in Reference [13], [14] are indicated by X. Statistics of the 3 positions under positive selection are represented in bold italics.

**Figure 2:**
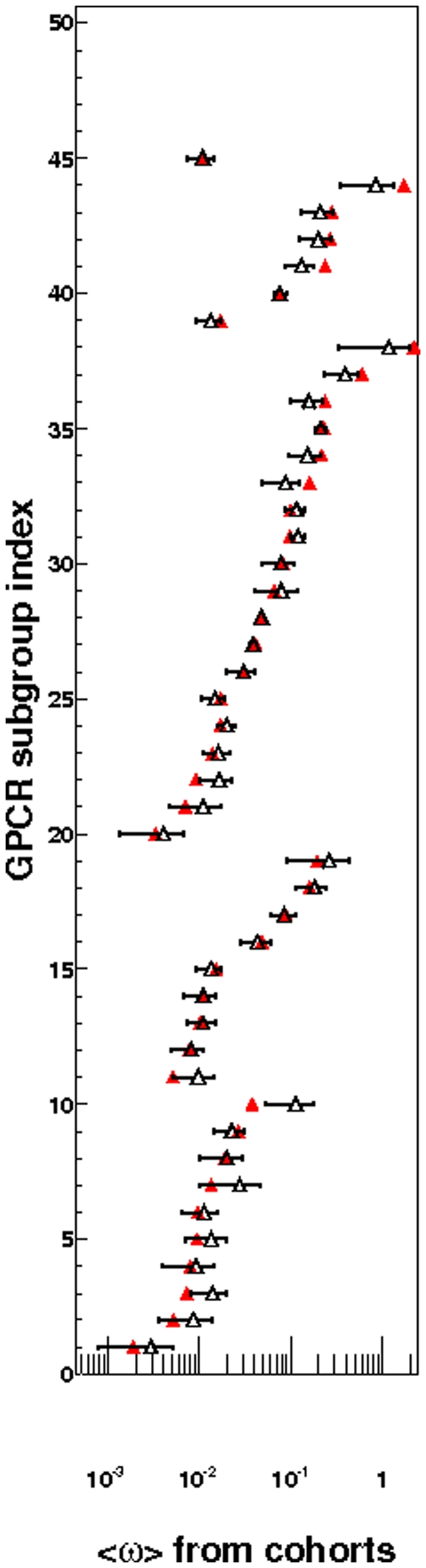
The average ω of key positions (<ω_key_>) contrasted with average ω of randomly selected 7TM positions (<ω_random7TM_>). Results of selection pressure, from PAML's model M7, for subgroups 1–45 and listed in Tables 1, 2, 3 and 4 are shown above. Results from model M8 were obtained for subgroups 38 and 44. Filled triangle represents <ω_key_> while open triangle represents the average of the average from random cohorts (from the <ω_random7TM_> distribution). The error bar represents two standard deviations (2σ_random7TM_) or the 95% confidence interval from ω_random7TM_ distribution.

**Figure 3:**
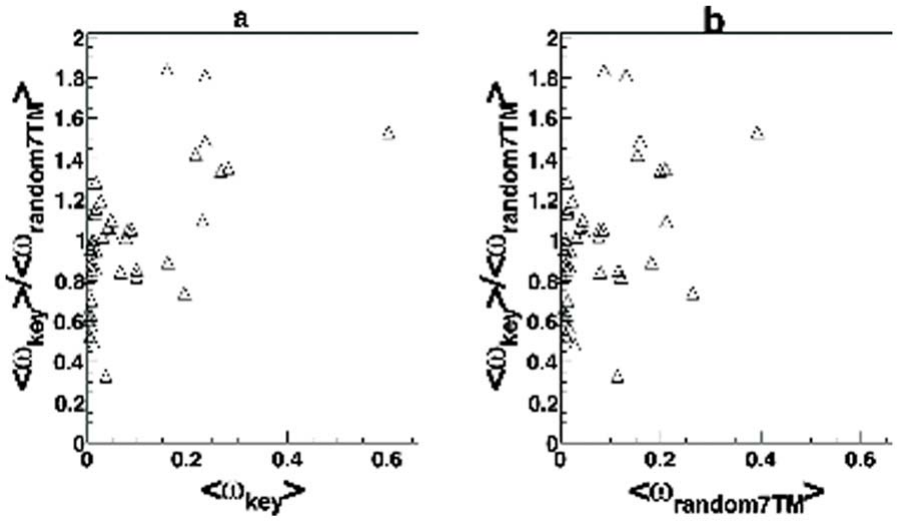
Graph showing the trends in <ω_key_>/<ω_random7TM_> vs. <ω>. Subgroups with pair-wise max(*d*_N_)<1 are represented in these panels. Subgroups 38 and 44 are excluded to avoid bias due to positive selection. **a**) Plot of <ω_key_>/<ω_random7TM_> vs. <ω_key_>. **b**) Plot of <ω_key_>/<ω_random7TM_> vs. <ω_random7TM_>.

We next compared <ω_key_> to random sets of 7TM positions <ω_random7TM_> to see if there was stronger selection pressure at the key positions. The values are shown in Figure 2 and Figure S1. For most receptor subgroups binding to small molecules, <ω_key_> was less than <ω_random7TM_> although within two standard deviations of <ω_random7TM_>. The selection pressure for subgroup 42 was atypical in that <ω_key_> was larger than <ω_random7TM_> by two standard deviations. For six of nine subgroups associated with lipid-activated receptors, <ω_key_> was nearly equal to <ω_random7TM_>. In subgroups activated by peptides, <ω_key_> was less than or nearly equal to <ω_random7TM_>. Subgroup 38, which exhibits strong positive selection, was the only other case where <ω_key_> exceeded <ω_random7TM_> by two standard deviations. Linear regression of <ω_key_> vs. <ω_random7TM_> for the subgroups excluding subgroup 38 and 44, showed a linear dependence (R^2^ = 0.892, p<2.2×10^−16^) (See Figure S2). However, as seen in Figure 3, <ω_key_>/<ω_random7TM_> is less than unity for small <ω_key_> and increases significantly with <ω_key_> (p<3.6×10^−6^) and <ω_random7TM_> (p<4.9×10^−3^). The dependence remained significant even after including subgroup 38. The Yang and Swanson's “fixed sites” model [25] indicated that <ω_key_> was significantly lower than <ω_random7TM_> in two of the ten small molecule subgroups (subgroups 3 and 10). Subgroup 11, which consists of lipid-activated receptors, showed statistically significant differences between key and random positions. In 5 of the 19 subgroups of the peptide receptors, key positions have significantly higher selection pressure then random positions. Only subgroup 22 of the peptide-activated receptors was significantly lower. The results are summarized in Table S1.

We also tested if the diversity of ω_key_ values in subgroups was due to the dissimilarity among amino acid (AA) residues at a given MSA position since it is expected that stronger selection pressure should result in lower variability. However, the strength of the correlation between ω_key_ and variability was not known. We examined this with three different measures. First, we computed the Shannon entropy (*H*) for the key positions of each subgroup, which has a theoretical range of 0 bits≤*H*≤4.32 bits. Figure S3 shows *H* for every key position across all subgroups. Figure S4 is a plot of H vs. <ω_key_> for subgroups with average pair-wise max(*d*_N_)<1 (see Materials and Methods). This figure shows a slight trend of higher entropy for higher <ω_key_> although it was not statistically significant. A linear regression of <*H*_key_> against log_10_<ω_key_> found a correlation coefficient of R = 0.47 (p<1.4×10^−3^). However, the regression of <*H*_key_> against log_10_ <ω_key_> had much lower correlation when <ω_key_> was restricted to <ω_key_> <0.1 (R = 0.26, p<9.8×10^−2^). However, this decrease in correlation could be due to the decrease in statistical power because the sample size is reduced. Similar results were found using the BLOSUM80 substitution matrix [26] and a distance matrix D_key_ to estimate the dissimilarity among residues within subgroups at key positions. Results are in Figures S5, S6, S7, and S8. These results show that AA variability at MSA positions is only weakly correlated with <ω_key_> and the correlation is weaker for subgroups under strong negative selection.

## Discussion

We have found that class A GPCR subgroups that are naturally activated by small molecules possessed strong negative selection in the key positions. Additionally, the selection pressure at the key positions is more likely to be stronger than the rest of the TM positions in small molecule receptors. The existence of strong negative selection supports coevolution over evolutionary drift as an explanation for the high mutual information between the key positions. We suggest that collective substitutions of key residues under strong selection pressure may have altered function in GPCRs. It has been shown previously that evolutionary characteristics such as phylogeny and sequence similarity of AA residues are a strong predictor of determinants of ligand specificity [27], [28], [29].

Under the rules of formal logic, the observation that small molecule receptors are always under strong negative selection at key positions allows for the prediction that GPCRs not under strong negative selection pressure are not naturally activated by small molecules. Based on our results from Figures 2 and S1, a threshold of ω = 0.1 can be established for strong negative selection (Figures 2 and S1 show that max(ω_key_≈0.05) and max(ω_random7TM_≈0.1)). We thus predict that receptor subgroups with ω>0.1 at the key positions do not possess a natural small molecule ligand. This would include orphan receptors MAS1L, MRGPRF of group 41, MRGPRX3, MRGPRX4 of 38 and 44, and TAAR5, TAAR6, TARR8, and TAAR9 of 42. The inclusion of subgroup 42 may be considered to be surprising because TAAR1 of the group binds β-phenylethylamine and p-tryamine, which is a small molecule trace amine. Although this subgroup exhibits negative selection in conformation of recent studies involving TAAR orthologs [30], [31] it is not strongly negative. This may imply that even though TAAR1 binds a trace amine, the key positions may not be vigorously maintaining their functionality.

**Figure 4:**
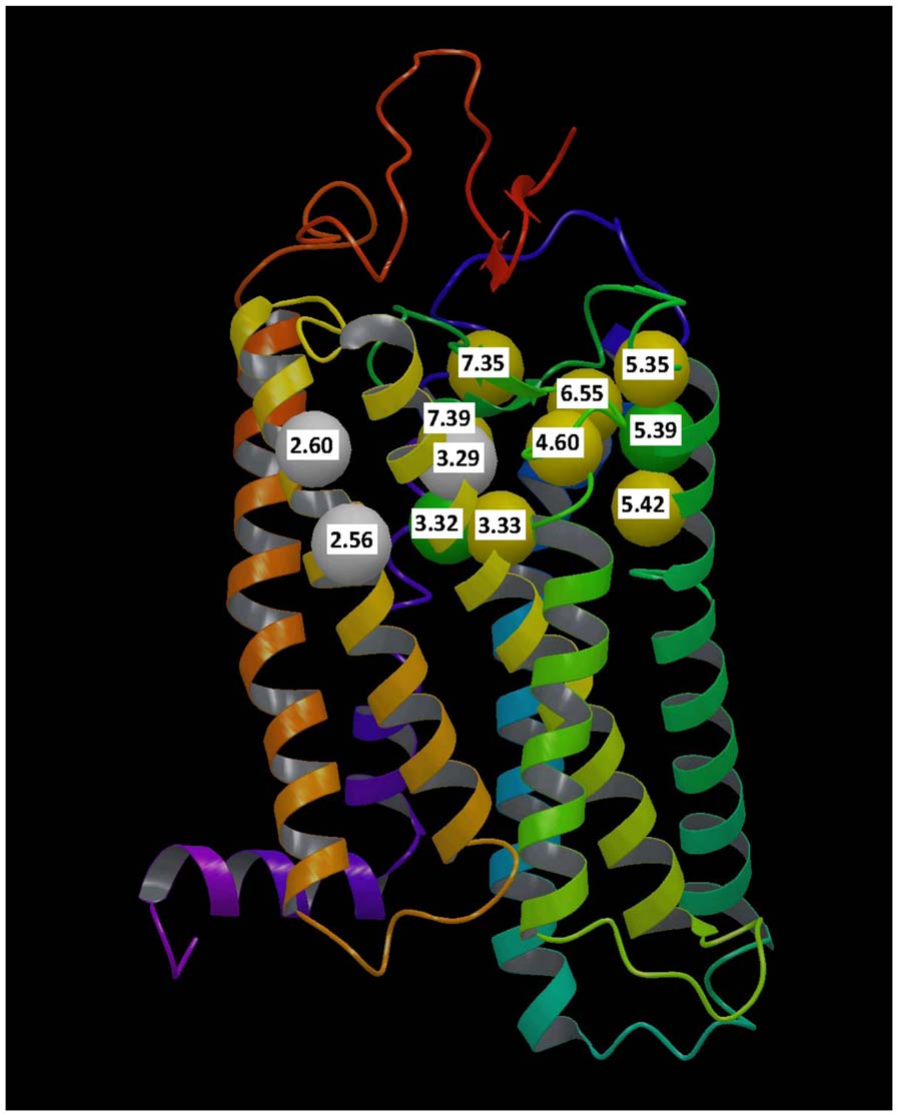
Notable positions of the MRGX1-type receptors visualized in the crystal structure of bovine rhodopsin. Positions 2.56, and 2.60 and 3.29 are under positive selection pressure and shown in white −3.29 is a key position, while 2.56 and 2.60 are not. Residues at two key positions, 3.32 and Val 204 5.39 are under negative selection pressure and shown in green. Residues at remaining 7 key positions are not under strong selective pressure are shown in yellow. Those positions are 3.33, 4.60, 5.35, 5.42, 6.55, 7.35 and 7.39. The figure is relative to the structure of bovine rhodopsin published by Schertler and coworkers (PDB ID: 1GZM) [70]. The notable positions are represented through spheres centered on the Cα atoms of the corresponding rhodopsin residues. The backbone of the receptor is schematically represented as a ribbon, colored with continuum spectrum that transitions from red to purple moving from the N-terminus to the C-terminus (TM1: dark orange; TM2: light orange; TM3: yellow; TM4: yellow/green; TM5: green; TM6: cyan; TM7: blue/purple).

Positive selection can lead to adaptation of a previous function [32], [33], [34], [35]. Strong statistical evidence for positive selection was identified at key position 3.29 of subgroup 38 but not for subgroup 41, both of which are composed of MAS-related GPCRs. Statistical evidence for positive selection at key position 3.29 was identified in subgroup 44, with decreased statistical significance (results not shown). Because subgroup 44 comprises of subgroups 41 (MAS1L, MRGD, MAS, MRGRF) and 38 (MRGX1, MRGX2, MRGX3, MRGX4), sustained positive selection at 3.29 suggests adaptation specific to subgroup 38. Notably, in the 3D crystal structure of bovine rhodopsin [36], positions 3.29, 2.56 and 2.60 are near neighbors when represented on the resolved crystal structure of bovine rhodopsin in Figure 4. This suggest that, if there has been any novel or adaptive function in the interhelical cavity of MRGX1-type receptors, then it may have evolved via mutations (substitutions) that occurred in that circumscribed region of the receptor. Therefore, as a continuation of our novel bioinformatic approach, we identified an AA position from a cohort of statistically related AA positions in a protein family (namely, class A GPCRs) that evolves under strong positive selective pressure in a subgroup (namely, subgroup 38).

We examined entropy and measures of sequence similarity to test the hypothesis that strong selection pressure is related to low variability. Our results showed that even under strong negative selection pressure, sequence diversity remained. The wide diversity in selection pressure for receptors associated with the different classes of natural ligands was not attributable to the size of the subgroup. Diversity of ω values is well documented [37], [38], [39], [40] and for the different subgroups of GPCRs may be attributed to differences in the (i) natural ligands they bind, (ii) molecular mechanism of activation, (iii) phylogeny of the subgroups, and (iv) ubiquity of expression on cell surfaces [41], [42], [43].

The inclusion of orthologs would improve the accuracy of our analysis. We used three overlapping subgroups: 13b (overlapping with 13), 43 (overlapping with 37) and 44 (overlapping with 38 and 41) to probe how ω_key_ and ω_random7TM_ changed with subgroup size. Subgroup 13b contained a pseudogene GPR42. Studies of class A GPCR orthologs have been previously investigated using opsins, MAS-related receptors, P2Y receptors and melanocortin receptors [22], [23], [44], [45], [46], [47], [48], [49], [50], [51]. Amongst the GPCRs we studied, statistically significant positive selection has been widely reported for visual opsin receptors (receptors for trichromatic vision in old world primates) and subgroup 38 of MAS-related receptors (receptors for pain and itch). The divergence among human GPCR subgroups is varied and high polymorphism may be seen from recent studies, e.g. in the case of human MRGX1 receptors [52].

## Materials and Methods

### Identification of key positions

An alignment of human non-olfactory class A 7TMs was obtained from [53]. Using that MSA, we identified a clique of statistically related MSA positions. These key positions had the highest collective MI with respect to one another and most other positions in the MSA [13], [14]. The Ballesteros-Weinstein indexing scheme for GPCRs [24] was used to label all positions of the MSA.

### Input data – nucleotide sequence data corresponding to 7TMs

Nucleotide sequence fragments that encoded the GPCR 7TMs were obtained from NCBI's nucleotide database [54]. The cDNA sequence records encoding the entire protein sequence was extracted using NCBI's Open Reading Frame online resource [55]. Entire AA sequence records were obtained from the RefSeq database [56] and the Uniprot database [57]. The amino acid and nucleotide sequence fragments from the 7TMs were concatenated. We used the IUPHAR 7TM receptor database [58], [59] as well as a comprehensive GPCR listing from Gloriam et al. [60] to confirm our sequence data.

### Input data – Phylogenetic tree

We used AA sequence fragments for the 7TMs of class A GPCRs to reconstruct a nearest neighbor phylogenetic tree. Program PROTDIST of PHYLIP [61] was used to compute phylogenetic distance across pairs of concatenated 7TM fragments using the JTT matrix for AA substitutions [62]. The nearest neighbor joining method [63] implemented in PHYLIP's program NEIGHBOR was used to reconstruct the tree. Subgroups of GPCRs representing closely related 7TMs were identified from the phylogenetic tree, using a bootstrap approach. The selection of subgroup was refined using *d*_N_ and *d*_S_ selection criteria described below. A consensus phylogenetic tree was obtained using the CONSENSE program of PHYLIP. A list of GPCRs for all subgroups is shown in Tables 1, 2, 3 and 4.

### GPCR subgroups

We analyzed forty-five subgroups, of which forty-two were non-overlapping and distinct. The number of constituent GPCRs in respective subgroups ranged from three to ten. Because GPCRs are highly divergent, we restricted the average maximum *d*_N_ and maximum *d*_S_ estimated from all pairs of receptors within subgroups unlike in a traditional analysis where subgroups may be clearly identified as distinct clades from a familial phylogenetic tree. We used the counting scheme of Nei-Gojobori to estimate the average *d*_N_ and *d*_S_ from pairs of sequences [64]. We investigated subgroups where the maximum average *d*_N_ of all pair-wise comparisons within the subgroup did not exceed 1. If the condition of max(*d*_N_)<1 was not met, then the out group taxa was removed, and the subgroup reduced. There was no *a priori* scheme to identify subgroups to achieve the max(*d*_N_) and max(*d*_S_) conditions. To study the measurement uncertainties due to sample size, we analyzed subgroups having progressively larger numbers of closely related receptors. The subgroups in which it exceeded 1 were indicated by “N” in Tables 1, 2, 3 and 4 and were not included in Figure S4, Figure S6 and Figure S8. We found that max(*d*_N_)<1 selection resulted in max(*d*_S_)<3 for forty of forty-five subgroups. Subgroups listed in Tables 1, 2, 3 and 4 and denoted by “S” did not meet max(*d*_S_)<3. The d*_N_* and d*_S_* obtained after maximum likelihood computation was more conservative compared to that obtained via the Nei-Gojobori counting method (results not shown).

### Estimation of ω at AA positions across 7TMs

PAML version 4.2b [65] was used to model the evolution of the 7TM nucleotide sequences using a state space of possible codons from the genetic code. The program simulated the molecular evolution of the concatenated 7TM fragments independently, for each subgroup. Four independent strategies from PAML were used to estimate ω. Two mathematical models were tested for statistical tenability in each strategy. The constraints and assumptions for estimating ω were accommodated differently in the models. In the first strategy, model M2a accommodated positions under negative selection via ω = ω_0_ (ω_0_<1), a free parameter determined from data, that was common for most 7TM positions. In addition, to represent neutral evolution, a portion of the remaining 7TM positions were constrained to ω_1_ = 1. Lastly, with another free parameter, the same model also accommodated representation of positive selection for the remaining fraction of positions (ω_2_>1). In contrast, model M1a was a special case of M2a, in which it excluded positive selection. Because ω for an AA position under near-neutral evolution was also constrained to unity, this was the most conservative of the three strategies. Test 1 compares M1a vs. M2a.

In the second strategy the spectrum of ω values from MSA positions was represented by a beta function (with two free parameters *p* and *q*). Model M8 represented the spectrum of ω across all MSA positions with ten discrete ω_i_ categories to represent the beta function (for ω_i_≤1, i = 0,1,2…,9). An additional eleventh category ω_10_ accounted for a small fraction of positions under positive selection. In model M7, there was no provision for such positive selection (p_10_ = 0, therefore ω_10_ was absent). Test 2 compares M7 vs. M8.

In a third strategy, we used Yang and Swanson's “fixed sites” models A and B [25]. The null model (model A) hypothesized that there was no statistically distinct selection pressure among the MSA positions. We used the simplest alternate model (model B), from the suite of “fixed sites” models, which hypothesized that the average evolutionary selection pressure from cohort of key MSA positions was statistically distinct with respect to the other MSA positions.

In all the three strategies, which we refer to as Tests 1–3 in Table S1, a maximum likelihood ratio test was used to determine the tenable model from competing nested paired models. The goal of both models was to represent the observed evolutionary data – the MSA of nucleotide 7TM sequences and the phylogenetic tree from the relevant subgroup. In each strategy, the maximum likelihood of the null model M_Null_ that could fit the data was compared with that obtained from an alternate model M_Alt_ (which had additional free parameters compared to the null model).

In a fourth strategy, which we called Test 4, model M3 was compared to model M0 for all subgroups. The alternative model demonstrated the heterogeneity of ω values across the 7TMs and the null model was representative of their common ω value. Test 4 is not specific for inferring positive selection and all results are shown in Table S2.

### Chemical class of the natural ligands associated with class A GPCRs

Subgroups were classified into three categories in terms of their natural ligands: 1) small molecules (including biogenic amines, nucleosides and nucleotides), 2) lipids and 3) peptides. If subgroups did not exclusively bind the same chemical class of natural ligand or if they had more than two orphan receptors, then we categorized them as divergent. If subgroups exclusively contained orphan receptors then they were categorized as orphan.

### Computing average ω from randomly selected 7TM AA positions

To compare <ω_key_> with randomly selected 7TM positions, two hundred cohorts of AA positions were simulated. The average ω from each of the cohort of ten randomly selected 7TM positions was computed – this was denoted as <ω_random7TM_>. The average of the two hundred independent cohorts was computed from the distribution of <ω_random7TM_>.

### Computing AA diversity at key positions

Shannon entropy was first used to estimate the diversity in AA composition at key positions across all subgroups. The Shannon entropy at MSA position *X,* with AA residues *x,* was defined as
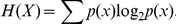
Here the summation is over all rows *r* of the MSA, *p*(*x*) was the probability of having residue *x* at position *X*, and the summation is over all AA residues.

A variety of strategies exist to quantify sequence similarity [66]. We used two independent approaches to estimate the similarity of key AA residues using all subgroups. In the first method, sequence similarity was estimated with the BLOSUM substitution matrix [26]. Consider *S* to be the number of concatenated 7TM fragments in a subgroup. The AA similarity (and dissimilarity) among MSA positions of 7TM fragments due to substitutions among the *S* different paralogs of the subgroup was determined. We used BLOSUM80 substitution matrix to evaluate sequence similarity among the residues at key positions of the MSA. For a given key position, the average score of the key AA residues substituting with each other within the subgroup, we used the definition of Karlin and Brocchieri [67], given by the equation
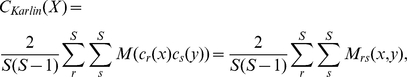
where *c_r_*(*x*) is the AA at MSA position (or column) *X* in the *s*th fragment, and *M_rs_*(*x*,*y*) scores for substitution between AA *x* and AA *y*. This similarity score *M_rs_*(*x,y*), for the defined (*r*,*s*) pairs of AAs in the *r* ^th^ and *s* ^th^ sequence fragment, is defined as
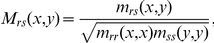
where *m_rs_*(*x,y*) is the BLOSUM80 [26] matrix element corresponding to substitution from AA *x* in the *r* ^th^ row to AA *y* in the *s* ^th^ row of the alignment (or vice versa). We defined the BLOSUM similarity score for a given key position *X* as *BLO_80_key_ = C_Karlin_*(*X*), and the average similarity score of all key positions <*BLO_80_key_*> was averaged over the ten key positions.

In another approach, another estimate for dissimilarity was obtained using residues from MSA columns at key positions. To represent a distance measure, the average percentage of accepted mutation using program PROTDIST from PHYLIP software [61] was obtained for all key positions in subgroups. That measure was denoted as D_key_. The quantity −log_10_(D_key_) was computed to compare the attribute with previously computed measures of sequence similarity.

## Supporting Information

Figure S1 **The average ω from key positions (<ω_key_>) contrasted with average ω from randomly selected 7TM positions (<ω_random7TM_>).** Results of selection pressure, from PAML's model M7 vs. M8, for subgroups 1–45, as listed in Tables 1, 2, 3 and 4 of manuscript, are shown. The ω values on the Y axis are represented in a linear scale (panel A) and logarithmic scale (panel B – Figure 2 in manuscript). Subgroups from 1–10 (shown in Table 1) are receptors naturally activated by small molecules, 11–19 (shown in Table 2) by lipids and 20–38 (shown in Table 3) by peptides. Subgroups 39–44 (shown in Table 4) are divergent. Subgroup 45 exclusively contains orphan GPCRs. Filled (red colored) triangle represents <ω_key_> while open triangle represents the average from random cohorts (from <ω_random7TM_> distribution). The error bar represents two standard deviations (2σ_random7TM_) or the limits of 95% confidence interval from ω_random7TM_ distribution.
(TIFF)

Figure S2 **Graph of <ω_key_> vs. <ω_random7TM_>.** Trend from <ω_key_> vs. <ω_random7TM_> is shown using a logarithmic scale. Graph excludes subgroups labeled as “N” in Tables 1, 2, 3 and 4 and excludes subgroups 38 and 44.
(TIFF)

Figure S3 **Shannon entropy (** ***H*** **) for key positions across GPCR subgroups.**
(TIFF)

Figure S4 **Average Shannon entropy vs. average selection pressure for key positions across subgroups.** Average scores from Figure S3 are plotted along the Y axis. Average evolutionary selection pressure from Figure 1 is represented using a logarithmic scale on the X axis. Subgroups not labeled “N” from Tables 1, 2, 3 and 4 (having pair-wise max(*d*_N_)<1) are represented here.
(TIFF)

Figure S5 **Similarity scores for key positions across GPCR subgroups.** Similarity scores (<BLO_80_key_>) in subgroup MSA defined by Karlin and Brocchieri, as in Reference 67, (described in Materials and Methods) generated using BLOSUM80 matrix.
(TIFF)

Figure S6 **Average similarity score <BLO_80_key_> vs. average selection pressure for key positions across subgroups.** Average scores from Figure S5 are plotted along the Y axis. Average evolutionary selection pressure from Figure 1 is represented using a logarithmic scale on the X axis. Subgroups not labeled “N” from Tables 1, 2, 3 and 4 (having pair-wise max(*d*_N_)<1) are represented here.
(TIFF)

Figure S7 **Inverse protdist distance measure (<−log_10_D_key_>) for key positions across GPCR subgroups.** Plot showing the logarithm of inverse protdist distance (D) at key positions from GPCR subgroups.
(TIFF)

Figure S8 **Average inverse protdist distance vs. average selection pressure for key positions across subgroups.** The Y-axis represents <−log_10_D_key_> from Figure S7. Average evolutionary selection pressure is represented using a logarithmic scale on the X axis. Subgroups not labeled “N” from Tables 1, 2, 3 and 4 (having pair-wise max(*d*_N_)<1) are represented here.
(TIFF)

Table S1 **Tenable PAML models representing molecular evolution of 7TMs of class A non-olfactory human GPCR subgroups.** PAML's tenable models that represent molecular evolution of their 7TMs are illustrated across GPCR subgroups. Results from two “random sites models” M2a vs.M1a (Test 1), M8 vs. M7 (Test 2) and that from Yang-Swanson “fixed sites” model A vs. model B (Test 3) are presented in columns 5–7. Tenable alternative models are represented “A” and tenable null models labeled “-”. Bold font in column 3 connotes orphan GPCR. Bold and italics font in columns 5–7 connote inference of positive selection.
(DOC)

Table S2 **Tenable PAML models representing molecular evolution of 7TMs of class A non-olfactory human GPCR subgroups.** PAML's tenable models that represent molecular evolution of their 7TMs are illustrated across GPCR subgroups. Results from “random sites models” M3 vs. M0 (Test 1) are presented. Tenable alternative models are represented “A” and tenable null models labeled “-”. Bold font in column 3 connotes orphan GPCR. Bold and italics font in columns 5 connotes inference of significant positive selection.
(DOC)
