# Supplementary figures and images for: Molecular Evolution of the Transmembrane Domains of G Protein-Coupled Receptors
Source: PLoS One. 2011 Nov 21;6(11):e27813. doi: 10.1371/journal.pone.0027813 (PMC3221663; doi:10.1371/journal.pone.0027813)

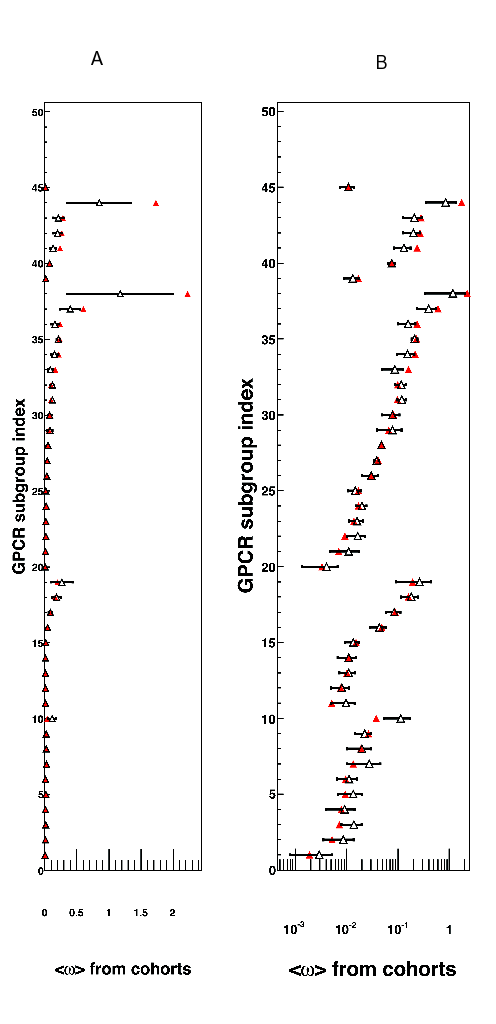

Supplement: Figure S1 — The average ω from key positions (<ωkey>) contrasted with average ω from randomly selected 7TM positions (<ωrandom7TM>). Results of selection pressure, from PAML's model M7 vs. M8, for subgroups 1–45, as listed in Tables 1, 2, 3 and 4 of manuscript, are shown. The ω values on the Y axis are represented in a linear scale (panel A) and logarithmic scale (panel B – Figure 2 in manuscript). Subgroups from 1–10 (shown in Table 1) are receptors naturally activated by small molecules, 11–19 (shown in Table 2) by lipids and 20–38 (shown in Table 3) by peptides. Subgroups 39–44 (shown in Table 4) are divergent. Subgroup 45 exclusively contains orphan GPCRs. Filled (red colored) triangle represents <ωkey> while open triangle represents the average from random cohorts (from <ωrandom7TM> distribution). The error bar represents two standard deviations (2σrandom7TM) or the limits of 95% confidence interval from ωrandom7TM distribution. (TIFF) [file pone.0027813.s001.tiff]

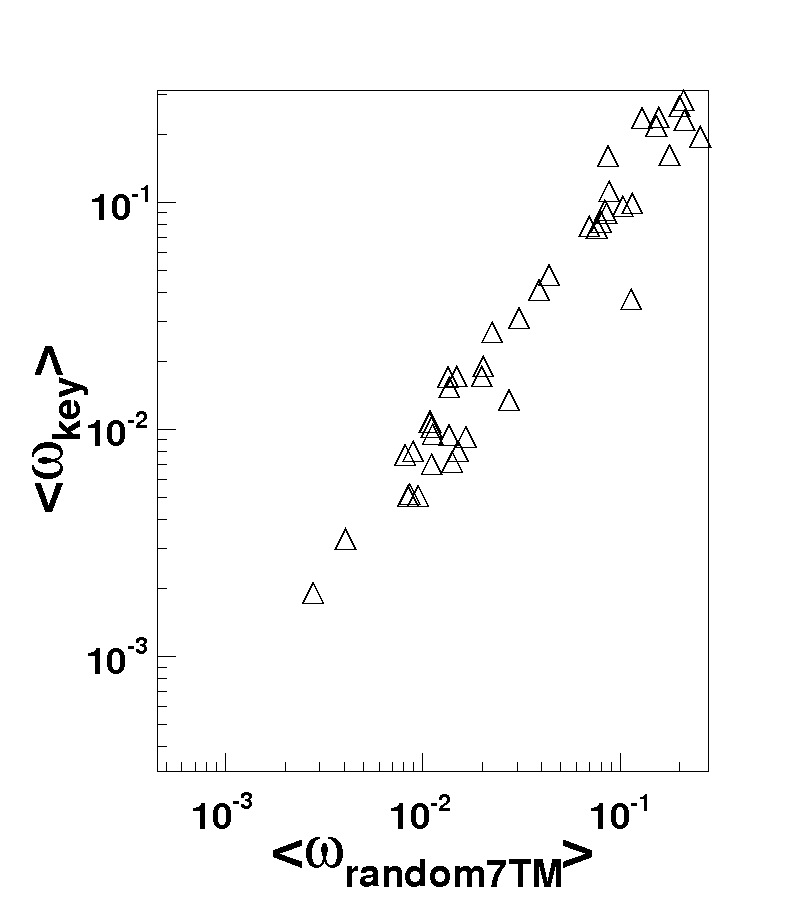

Supplement: Figure S2 — Graph of <ωkey> vs. <ωrandom7TM>. Trend from <ωkey> vs. <ωrandom7TM> is shown using a logarithmic scale. Graph excludes subgroups labeled as “N” in Tables 1, 2, 3 and 4 and excludes subgroups 38 and 44. (TIFF) [file pone.0027813.s002.tiff]

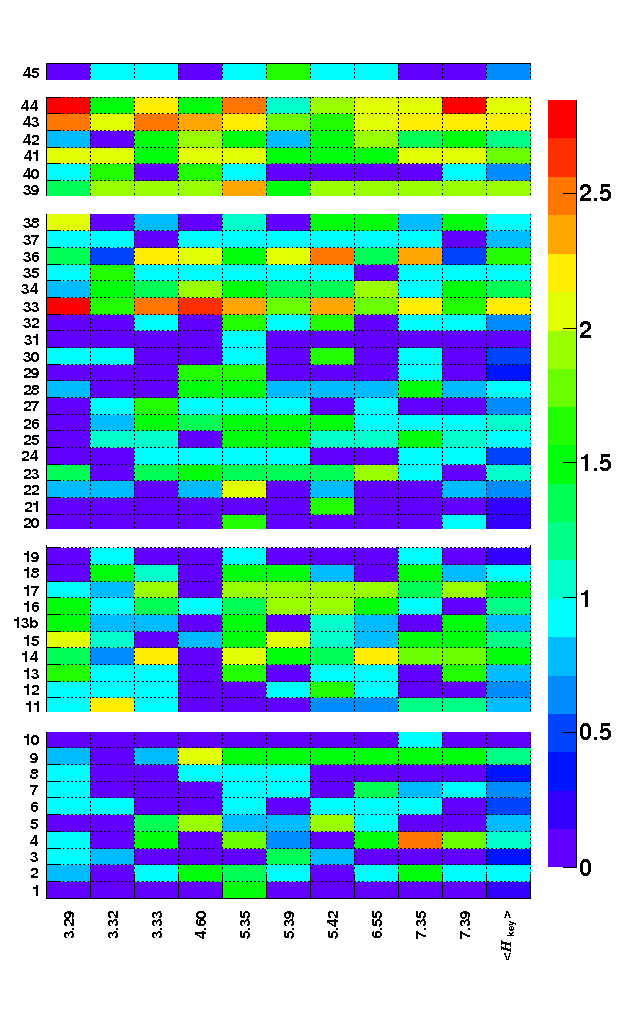

Supplement: Figure S3 — Shannon entropy ( H ) for key positions across GPCR subgroups. (TIFF) [file pone.0027813.s003.tiff]

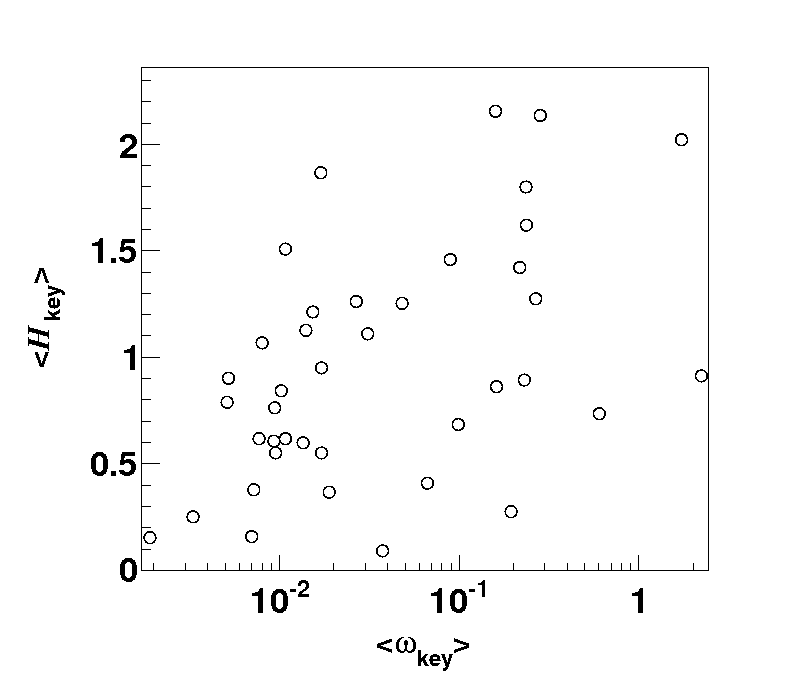

Supplement: Figure S4 — Average Shannon entropy vs. average selection pressure for key positions across subgroups. Average scores from Figure S3 are plotted along the Y axis. Average evolutionary selection pressure from Figure 1 is represented using a logarithmic scale on the X axis. Subgroups not labeled “N” from Tables 1, 2, 3 and 4 (having pair-wise max(d N)<1) are represented here. (TIFF) [file pone.0027813.s004.tiff]

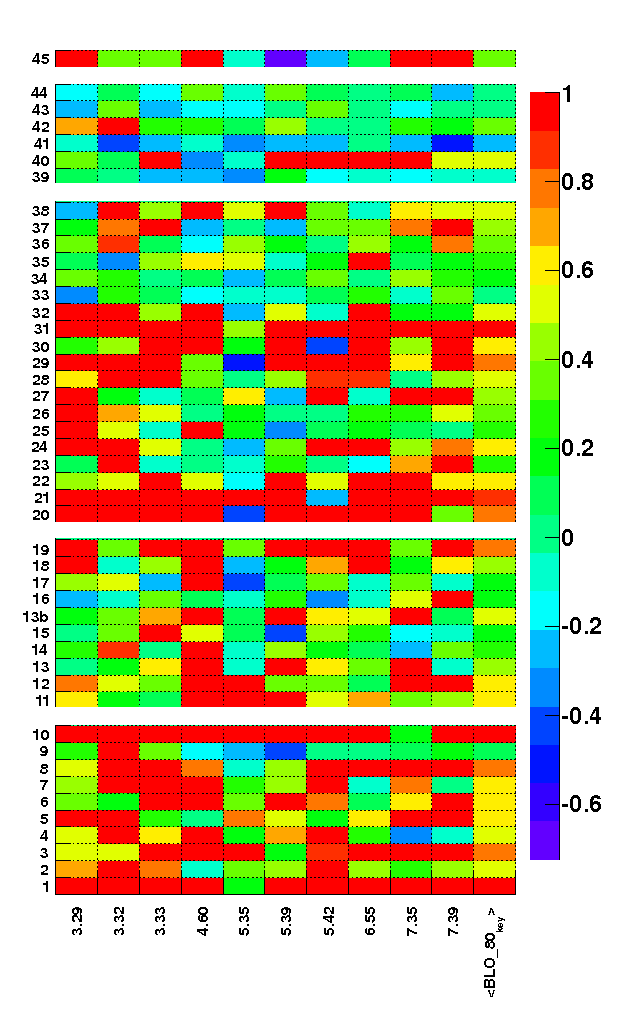

Supplement: Figure S5 — Similarity scores for key positions across GPCR subgroups. Similarity scores () in subgroup MSA defined by Karlin and Brocchieri, as in Reference 67, (described in Materials and Methods) generated using BLOSUM80 matrix. (TIFF) [file pone.0027813.s005.tiff]

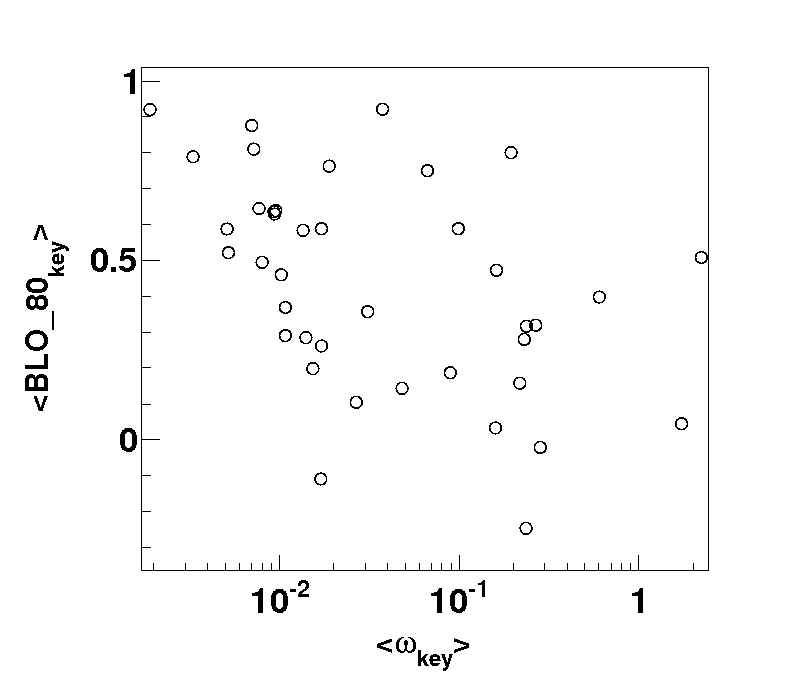

Supplement: Figure S6 — Average similarity score vs. average selection pressure for key positions across subgroups. Average scores from Figure S5 are plotted along the Y axis. Average evolutionary selection pressure from Figure 1 is represented using a logarithmic scale on the X axis. Subgroups not labeled “N” from Tables 1, 2, 3 and 4 (having pair-wise max(d N)<1) are represented here. (TIFF) [file pone.0027813.s006.tiff]

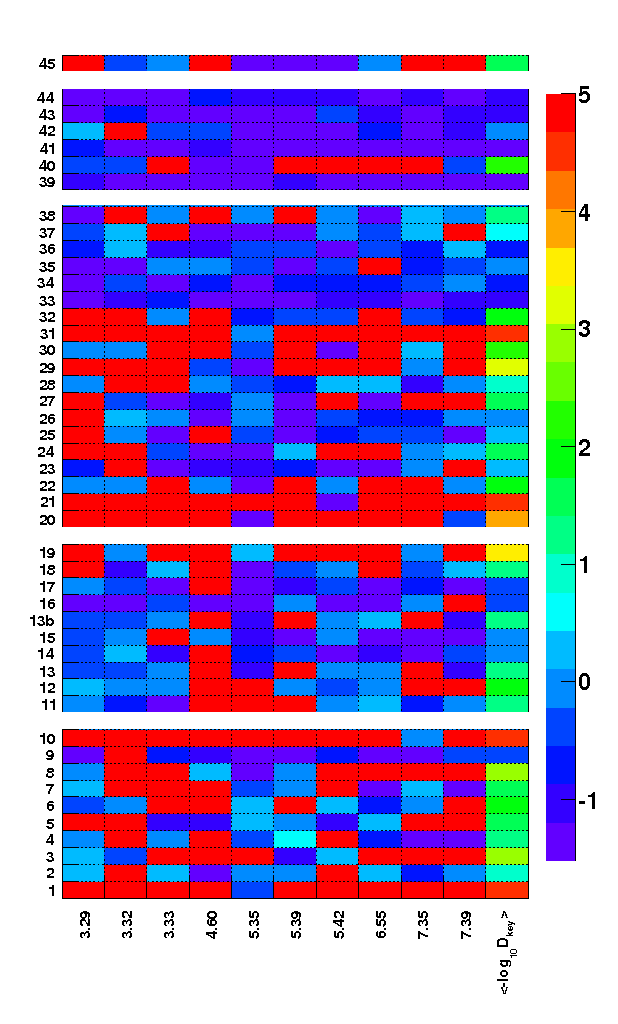

Supplement: Figure S7 — Inverse protdist distance measure (<−log10Dkey>) for key positions across GPCR subgroups. Plot showing the logarithm of inverse protdist distance (D) at key positions from GPCR subgroups. (TIFF) [file pone.0027813.s007.tiff]

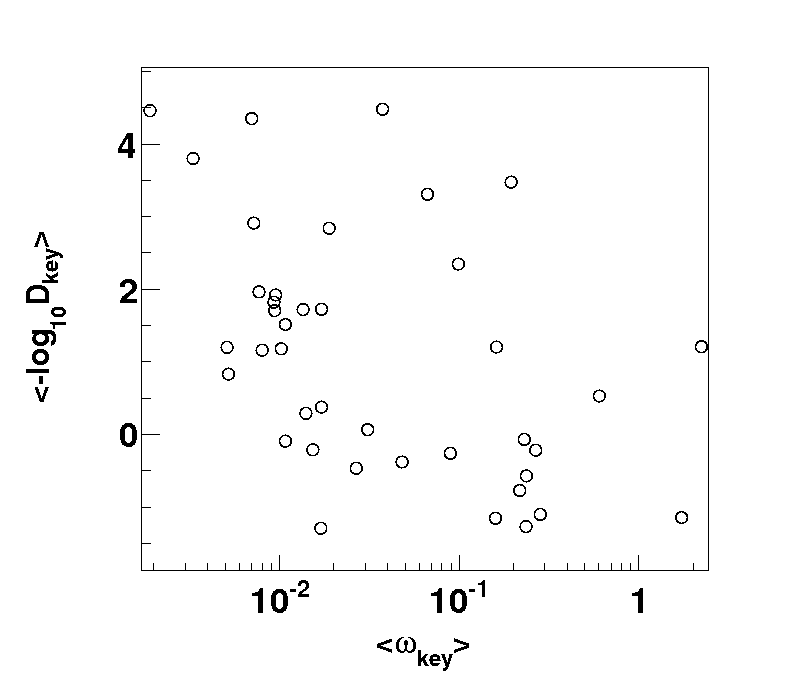

Supplement: Figure S8 — Average inverse protdist distance vs. average selection pressure for key positions across subgroups. The Y-axis represents <−log10Dkey> from Figure S7. Average evolutionary selection pressure is represented using a logarithmic scale on the X axis. Subgroups not labeled “N” from Tables 1, 2, 3 and 4 (having pair-wise max(d N)<1) are represented here. (TIFF) [file pone.0027813.s008.tiff]
