# Supplementary material for: Molecular Evolution of the Transmembrane Domains of G Protein-Coupled Receptors
Source: PLoS One. 2011 Nov 21;6(11):e27813. doi: 10.1371/journal.pone.0027813 (PMC3221663; doi:10.1371/journal.pone.0027813)
Supplement: Table S1 — Tenable PAML models representing molecular evolution of 7TMs of class A non-olfactory human GPCR subgroups. PAML's tenable models that represent molecular evolution of their 7TMs are illustrated across GPCR subgroups. Results from two “random sites models” M2a vs.M1a (Test 1), M8 vs. M7 (Test 2) and that from Yang-Swanson “fixed sites” model A vs. model B (Test 3) are presented in columns 5–7. Tenable alternative models are represented “A” and tenable null models labeled “-”. Bold font in column 3 connotes orphan GPCR. Bold and italics font in columns 5–7 connote inference of positive selection. (DOC) [file pone.0027813.s009.doc]

**Table S1. Tenable PAML models representing molecular evolution of 7TMs of class A non-olfactory human GPCR subgroups.** PAML’s tenable models that represent molecular evolution of their 7TMs are illustrated across GPCR subgroups. Results from two “random sites models” M2a vs.M1a (Test 1), M8 vs. M7 (Test 2) and that from Yang-Swanson “fixed sites” model A vs. model B (Test 3) are presented in columns 5-7. Tenable alternative models are represented “A” and tenable null models labeled “-”. Bold font in column 3 connotes orphan GPCR. Bold and italics font in columns 5-7 connote inference of positive selection.

| **Subgrp idx** | **# GPCRs in subgrp** | **GPCRs included in the subgroups** | **Chemical class of natural ligand** | **Test 1**  **tenable model** | **Test 2 tenable model** | **Test 3 tenable model** |
| --- | --- | --- | --- | --- | --- | --- |
| 1 | 5 | CHRM1 (ACM1), CHRM2 (ACM2), CHRM3 (ACM3), CHRM4 (ACM4), CHRM5 (ACM5) | small | - | - | - |
| 2 | 5 | DRD1, DRD2, DRD3, DRD4, DRD5 | small | - | - | - |
| 3 | 5 | P2RY12 (P2Y12), P2RY13 (P2Y13), P2RY14 (P2Y14), GPR87, **GPR171** (**GP171**) | small | - | - | A |
| 4 | 7 | HTR1A (5HT1A), HTR1B (5HT1B), HTR1D (5HT1D), 5HT1F (HTR1F), HTR1E (5HT1E) , HTR5A (5HT5A), HTR7 (5HT7R) | small | - | - | - |
| 5 | 5 | P2RY1, P2RY2, P2RY4, P2RY6, P2RY11 (P2Y11) | small | - | - | - |
| 6 | 3 | MTNR1A (MTR1A), MTNR1B (MTR1B), **GPR50** (**MTR1L**) | small | - | - | - |
| 7 | 5 | ADRA1A (ADA1A), ADRA1B (ADA1B), ADRB1, ADRB2, ADRB3 | small | - | - | - |
| 8 | 3 | HTR2A (5HT2A), HTR2B (5HT2B), HTR2C (5HT2C) | small | - | - | - |
| 9 | 4 | HRH1, HRH2, HRH3, HRH4 | small | - | - | - |
| 10 | 3 | ADORA1 (AA1R), ADORA2A (AA2AR), ADORA2B (AA2BR) | small | - | - | A |
|  |  |  |  |  |  |  |
| 11 | 6 | S1PR2 (EDG5), S1PR1 (EDG1), S1PR3 (EDG3), S1PR5 (EDG8), LPAR1 (EDG2), LPAR3 (EDG7) | lipid | - | - | A |
| 12 | 3 | GPR3, GPR6, GPR12 | lipid | - | - | - |
| 13 | 3 | FFAR1 (GPR40), FFAR2 (GPR43), FFAR3 (GPR41) | lipid | - | - | - |
| 14 | 7 | PTGDR (PD2R), PTGER1 (PE2R1), PTGER3 (PE2R3), PTGER4 (PE2R4), PTGFR (PF2R), PTGIR (PI2R), TBXA2R (TA2R) | lipid | - | - | - |
| 15 | 3 | CYSLTR1 (CLTR1), CYSLTR2(CLTR2), GPR17 | lipid | - | - | - |
| 13b | 4 | FFAR1 (GPR40), FFAR2 (GPR43), FFAR3 (GPR41), GPR42 (pseudogene) | lipid | - | - | - |
| 16 | 5 | LPAR4 (P2RY9), LPAR6 (P2RY5), **GPR174** (**GP174**), P2RY10 (P2Y10), PTAFR | lipid | - | - | - |
| 17 | 5 | RRH (OPSX), OPN3, OPN4, OPN5, RGR | lipid | - | - | - |
| 18 | 4 | OPN1MW (OPSG), OPN1LW (OPSR), RHO (OPSD), OPN1SW (OPSB) | lipid | - | - | - |
| 19 | 3 | GPR81, GPR109B (G109B), GPR109A (G109A) | lipid | - | - | - |
|  |  |  |  |  |  |  |
| 20 | 3 | TACR1 (NK1R), TACR1 (NK2R), TACR3 (NK3R) | peptide | - | - | - |
| 21 | 3 | TSHR, LHCGR (LSHR), FSHR | peptide | - | - | - |
| 22 | 4 | F2R (PAR1), F2RL1 (PAR2), F2RL2 (PAR3), F2RL3 (PAR4) | peptide | - | - | A |
| 23 | 5 | **GPR83**, NPY1R, NPY2R, PPYR1 **(**NPY4R), NPY5R | peptide | - | - | - |
| 24 | 3 | C3AR1 (C3AR), C5AR1 (C5AR), GPR77 (C5ARL) | peptide | - | - | - |
| 25 | 4 | EDNRA, EDNRB, **GPR37**, **GPR37L1** (**ETBR2**) | peptide | - | - | - |
| 26 | 5 | **LGR5**, **LGR6**, RXFP1 (LGR7), RXFP2 (LGR8) | peptide | - | - | - |
| 27 | 3 | GALR1, GALR2, GALR3 | peptide | - | - | - |
| 28 | 4 | OPRL1 (OPRX), OPRM1 (OPRM), OPRD1 (OPRD), OPRK1 (OPRK) | peptide | - | - |  |
| 29 | 3 | SSTR2 (SSR2), SSTR3 (SSR3), SSTR5 (SSR5) | peptide | - | - | - |
| 30 | 3 | GRPR, NMBR, BRS3 | peptide | - | - | - |
| 31 | 3 | MC3R, MC4R, MC5R | peptide | - | - | - |
| 32 | 3 | AVPR1A (V1AR), AVPR1B (V1BR), AVPR2 (V2R) | peptide | - | - | - |
| 33 | 10 | CXCR1, CXCR2, CXCR3, CXCR4, CXCR5, CXCR6, CCR6, CCR7, CCR9, CCR10 | peptide | - | - | A |
| 34 | 5 | APLNR (APJ), AGTR1 (AG2R, AG2S), RL3R1 (RLN3R2), RXFP4 (RLN3R2) | peptide | - | - | A |
| 35 | 3 | NTSR1 (NTR1), NTSR2 (NTR2), GPR39 | peptide | - | - | - |
| 36 | 9 | CCR1, CCR2, CCR3, CCR4, CCR5, CCR8, CCRL2, CX3CR1(CX3CR1, C3X1), CCBP2 | peptide | - | - | A |
| 37 | 3 | FPR1, FPR2 (FPRL1), FPR3 (FPRL2) | peptide | - | - | A |
| 38 | 4 | MRGPRX1 (MRGX1), MRGPRX2 (MRGX2), **MRGPRX3** (**MRGX3**), **MRGPRX4** (**MRGX4**) | peptide | ***A*** | ***A*** | ***A*** |
|  |  |  |  |  |  |  |
| 39 | 5 | **GPR101** (**GP101**), **GPR161** (**GP161**), **GPR135** (**GP135**), GPR63, **GPR45** | divergent | - | - | A |
| 40 | 3 | GPR4, GPR65 (PSYR), GPR68 (OGR1) | divergent |  |  |  |
| 41 | 4 | MAS1 (MAS), **MAS1L** (**MRG**), MRGPRD (MRGRD), **MRGPRF** (**MRGRF**, **GPR140**) | divergent | - | - | A |
| 42 | 5 | TAAR1 (TAR01), **TAAR5**, **TAAR6** (**TAR4**), **TAAR8** (**TAR5**), **TAAR9** (**TAR3**) | divergent | - | - | - |
| 43 | 10 | C3AR1 (C3AR), C5AR1 (C5AR), GPR77 (C5ARL), CMKLR1(CML1), FPR1, FPR2 (FPRL1), FPR3 (FPRL2), GPR1, GPR32, GPR44 (CRTH2) | divergent | - | - | A |
| 44 | 8 | MAS1 (MAS), **MAS1L** (**MRG**), MRGPRD (MRGRD), **MRGPRF** (**MRGRF**, **GPR140**), MRGPRX1 (MRGX1), MRGPRX2 (MRGX2), **MRGPRX3** (**MRGX3**), **MRGPRX4** (**MRGX4**) | divergent | ***A*** | ***A*** | ***A*** |
|  |  |  |  |  |  |  |
| 45 | 3 | **GPR27**, **GPR85**, **GPR173** | orphans | - | - | A |
