# Supplementary material for: Molecular Evolution of the Transmembrane Domains of G Protein-Coupled Receptors
Source: PLoS One. 2011 Nov 21;6(11):e27813. doi: 10.1371/journal.pone.0027813 (PMC3221663; doi:10.1371/journal.pone.0027813)
Supplement: Table S2 — Tenable PAML models representing molecular evolution of 7TMs of class A non-olfactory human GPCR subgroups. PAML's tenable models that represent molecular evolution of their 7TMs are illustrated across GPCR subgroups. Results from “random sites models” M3 vs. M0 (Test 1) are presented. Tenable alternative models are represented “A” and tenable null models labeled “-”. Bold font in column 3 connotes orphan GPCR. Bold and italics font in columns 5 connotes inference of significant positive selection. (DOC) [file pone.0027813.s010.doc]

**Table S2. Tenable PAML models representing molecular evolution of 7TMs of class A non-olfactory human GPCR subgroups.** PAML’s tenable models that represent molecular evolution of their 7TMs are illustrated across GPCR subgroups. Results from “random sites models” M3 vs. M0 (Test 1) are presented. Tenable alternative models are represented “A” and tenable null models labeled “-”. Bold font in column 3 connotes orphan GPCR. Bold and italics font in columns 5 connotes inference of significant positive selection.

| **Subgrp idx** | **# GPCRs in subgrp** | **GPCRs included in the subgroups** | **Chemical class of natural ligand** | **Test 4 tenable model** |
| --- | --- | --- | --- | --- |
| 1 | 5 | CHRM1 (ACM1), CHRM2 (ACM2), CHRM3 (ACM3), CHRM4 (ACM4), CHRM5 (ACM5) | small | A |
| 2 | 5 | DRD1, DRD2, DRD3, DRD4, DRD5 | small | A |
| 3 | 5 | P2RY12 (P2Y12), P2RY13 (P2Y13), P2RY14 (P2Y14), GPR87, **GPR171** (**GP171**) | small | A |
| 4 | 7 | HTR1A (5HT1A), HTR1B (5HT1B), HTR1D (5HT1D), 5HT1F (HTR1F), HTR1E (5HT1E) , HTR5A (5HT5A), HTR7 (5HT7R) | small | A |
| 5 | 5 | P2RY1, P2RY2, P2RY4, P2RY6, P2RY11 (P2Y11) | small | A |
| 6 | 3 | MTNR1A (MTR1A), MTNR1B (MTR1B), **GPR50** (**MTR1L**) | small | A |
| 7 | 5 | ADRA1A (ADA1A), ADRA1B (ADA1B), ADRB1, ADRB2, ADRB3 | small | A |
| 8 | 3 | HTR2A (5HT2A), HTR2B (5HT2B), HTR2C (5HT2C) | small | A |
| 9 | 4 | HRH1, HRH2, HRH3, HRH4 | small | A |
| 10 | 3 | ADORA1 (AA1R), ADORA2A (AA2AR), ADORA2B (AA2BR) | small | A |
|  |  |  |  |  |
| 11 | 6 | S1PR2 (EDG5), S1PR1 (EDG1), S1PR3 (EDG3), S1PR5 (EDG8), LPAR1 (EDG2), LPAR3 (EDG7) | lipid | A |
| 12 | 3 | GPR3, GPR6, GPR12 | lipid | A |
| 13 | 3 | FFAR1 (GPR40), FFAR2 (GPR43), FFAR3 (GPR41) | lipid | A |
| 14 | 7 | PTGDR (PD2R), PTGER1 (PE2R1), PTGER3 (PE2R3), PTGER4 (PE2R4), PTGFR (PF2R), PTGIR (PI2R), TBXA2R (TA2R) | lipid | A |
| 15 | 3 | CYSLTR1 (CLTR1), CYSLTR2(CLTR2), GPR17 | lipid | A |
| 13b | 4 | FFAR1 (GPR40), FFAR2 (GPR43), FFAR3 (GPR41), GPR42 (pseudogene) | lipid | A |
| 16 | 5 | LPAR4 (P2RY9), LPAR6 (P2RY5), **GPR174** (**GP174**), P2RY10 (P2Y10), PTAFR | lipid | A |
| 17 | 5 | RRH (OPSX), OPN3, OPN4, OPN5, RGR | lipid | A |
| 18 | 4 | OPN1MW (OPSG), OPN1LW (OPSR), RHO (OPSD), OPN1SW (OPSB) | lipid | A |
| 19 | 3 | GPR81, GPR109B (G109B), GPR109A (G109A) | lipid | A |
|  |  |  |  |  |
| 20 | 3 | TACR1 (NK1R), TACR1 (NK2R), TACR3 (NK3R) | peptide | A |
| 21 | 3 | TSHR, LHCGR (LSHR), FSHR | peptide | A |
| 22 | 4 | F2R (PAR1), F2RL1 (PAR2), F2RL2 (PAR3), F2RL3 (PAR4) | peptide | A |
| 23 | 5 | **GPR83**, NPY1R, NPY2R, PPYR1 **(**NPY4R), NPY5R | peptide | A |
| 24 | 3 | C3AR1 (C3AR), C5AR1 (C5AR), GPR77 (C5ARL) | peptide | - |
| 25 | 4 | EDNRA, EDNRB, **GPR37**, **GPR37L1** (**ETBR2**) | peptide | - |
| 26 | 5 | **LGR5**, **LGR6**, RXFP1 (LGR7), RXFP2 (LGR8) | peptide | A |
| 27 | 3 | GALR1, GALR2, GALR3 | peptide | - |
| 28 | 4 | OPRL1 (OPRX), OPRM1 (OPRM), OPRD1 (OPRD), OPRK1 (OPRK) | peptide | A |
| 29 | 3 | SSTR2 (SSR2), SSTR3 (SSR3), SSTR5 (SSR5) | peptide | A |
| 30 | 3 | GRPR, NMBR, BRS3 | peptide | A |
| 31 | 3 | MC3R, MC4R, MC5R | peptide | A |
| 32 | 3 | AVPR1A (V1AR), AVPR1B (V1BR), AVPR2 (V2R) | peptide | A |
| 33 | 10 | CXCR1, CXCR2, CXCR3, CXCR4, CXCR5, CXCR6, CCR6, CCR7, CCR9, CCR10 | peptide | A |
| 34 | 5 | APLNR (APJ), AGTR1 (AG2R, AG2S), RL3R1 (RLN3R2), RXFP4 (RLN3R2) | peptide | A |
| 35 | 3 | NTSR1 (NTR1), NTSR2 (NTR2), GPR39 | peptide | A |
| 36 | 9 | CCR1, CCR2, CCR3, CCR4, CCR5, CCR8, CCRL2, CX3CR1(CX3CR1, C3X1), CCBP2 | peptide | A |
| 37 | 3 | FPR1, FPR2 (FPRL1), FPR3 (FPRL2) | peptide | A |
| 38 | 4 | MRGPRX1 (MRGX1), MRGPRX2 (MRGX2), **MRGPRX3** (**MRGX3**), **MRGPRX4** (**MRGX4**) | peptide | ***A*** |
|  |  |  |  |  |
| 39 | 5 | **GPR101** (**GP101**), **GPR161** (**GP161**), **GPR135** (**GP135**), GPR63, **GPR45** | divergent | - |
| 40 | 3 | GPR4, GPR65 (PSYR), GPR68 (OGR1) | divergent |  |
| 41 | 4 | MAS1 (MAS), **MAS1L** (**MRG**), MRGPRD (MRGRD), **MRGPRF** (**MRGRF**, **GPR140**) | divergent | A |
| 42 | 5 | TAAR1 (TAR01), **TAAR5**, **TAAR6** (**TAR4**), **TAAR8** (**TAR5**), **TAAR9** (**TAR3**) | divergent | A |
| 43 | 10 | C3AR1 (C3AR), C5AR1 (C5AR), GPR77 (C5ARL), CMKLR1(CML1), FPR1, FPR2 (FPRL1), FPR3 (FPRL2), GPR1, GPR32, GPR44 (CRTH2) | divergent | A |
| 44 | 8 | MAS1 (MAS), **MAS1L** (**MRG**), MRGPRD (MRGRD), **MRGPRF** (**MRGRF**, **GPR140**), MRGPRX1 (MRGX1), MRGPRX2 (MRGX2), **MRGPRX3** (**MRGX3**), **MRGPRX4** (**MRGX4**) | divergent | ***A*** |
|  |  |  |  |  |
| 45 | 3 | **GPR27**, **GPR85**, **GPR173** | orphans | A |
